# Out-of-hospital cardiac arrest in Qatar: epidemiology, management, and outcomes from a national registry study

**DOI:** 10.1016/j.resplu.2025.101200

**Published:** 2025-12-22

**Authors:** Yavuz Yigit, Peter Alistair Cameron, Jassim Al Suwaidi, Loua Al Shaikh, Ibrahim Fawzy Hassan, Nidal Asaad, Nicholas Castle, Ian Lucas Howard, Abdulrahman Arabi, Atika Jabeen, Tim Richard Edmund Harris

**Affiliations:** a Department of Emergency Medicine, Hamad Medical Corporation, Doha, Qatar; b Blizard Institute, Queen Mary University, London, United Kingdom; c Cardiology Department, Heart Hospital, Hamad Medical Corporation, Doha, Qatar; d Major Incident Preparedness and Resilience & Ambulance Service Group, Hamad Medical Corporation, Doha, Qatar; e Medical Intensive Care Unit, Hamad Medical Corporation, Doha, Qatar; f Department of Medicine, Weill Cornell Medical College, Doha, Qatar

**Keywords:** Out-of-hospital cardiac arrest (OHCA), Resuscitation, Neurological outcomes, Return of spontaneous circulation, Bystander CPR, Qatar OHCA registry, Quality of life

## Abstract

**Background:**

Out-of-hospital cardiac arrest (OHCA) remains a major global health challenge with persistently low survival rates despite advances in resuscitation science. This study aimed to evaluate the epidemiology, management, and outcomes of OHCA in Qatar using a national registry aligned with Utstein reporting standards.

**Methods:**

A prospective observational cohort study was conducted across Qatar, enrolling all adult patients (≥18 years) with non-traumatic OHCA in whom resuscitation was attempted by the national EMS provider. Data were collected from EMS records, hospital EMRs, and mortuary databases. Survivors were followed up at 30 days and 12 months for neurological and quality-of-life outcomes. The primary outcome was 30-day survival with a favourable neurological status (CPC 1–2).

**Results:**

Among 1238 OHCA cases, the median age was 52 years, and 80.5 % were male. Arrests occurred predominantly at home (64.0 %), with 61.8 % witnessed and 42.4 % receiving bystander CPR. Initial shockable rhythms were present in 29.7 %. ROSC was achieved in 44.8 %, survival to discharge was 17.8 %, and a favourable neurological outcome at 30 days was 13.5 %. Multivariable analysis identified witnessed arrest, prehospital defibrillation, and coronary reperfusion within 24 h as independent predictors of survival. The Utstein comparator group demonstrated a survival rate of 38.2 % and CPC 1–2 outcome in 32.8 % of cases.

**Conclusions:**

OHCA outcomes in Qatar have improved markedly, with survival and CPC 1–2 rates more than doubling compared with prior national estimates. Survival now approaches levels seen in high-performing international systems, although within a younger patient population. Consistent predictors of outcome—including witnessed arrest, early defibrillation, and timely coronary reperfusion—emphasise the critical targets for strengthening OHCA systems of care.

## Introduction

Out-of-hospital cardiac arrest (OHCA) is a major public health problem; the incidence of EMS-treated OHCA is estimated at 73 per 100,000 population per year in the United States and 56 per 100,000 in Europe.^1^, ^2^ The median age for OHCA is in the 60 s, with a substantial proportion of these individuals having productive years ahead.^1^, ^3^, ^4^, ^5^, ^6^, ^7^ Although return of spontaneous circulation (ROSC) is achieved in around one quarter to two-thirds of cases, survival to hospital discharge remains low at 7–11 %.^4^, ^8^

Despite the introduction of targeted temperature management, active compression-decompression devices, impedance devices, emergent angioplasty, extracorporeal membrane oxygenation, and new pharmacologic therapies, overall survival rates have shown minimal improvement, with little change observed over the past 30 to 40 years.^8^, ^3^, ^4^, ^5^, ^9^ A recent systematic review concluded that *“we have a dearth of interventions that improve survival rates at hospital discharge and, even less so, neurological outcomes.”*^10^ While 70–80 % of survivors have a favourable neurological outcome (Cerebral Performance Category [CPC]1–2; modified Rankin Scale 0–3), neurologically intact survival across the total OHCA population remains low^7^, ^11^ at 5.8 % to 20.0 % in witnessed, shockable rhythms and 0.9–1.1 % in non-shockable rhythms.^12^, ^13^

In the past decade, studies from several countries have shown limited improvements in OHCA outcomes, with overall survival reported as high as 18 %.^4^, ^14^ The most important factors in improved survival are from prehospital care: improved rates of bystander cardiopulmonary resuscitation, increasingly rapid access to defibrillation and improved ambulance response times^15^, ^16^, ^17^ The Utstein comparator group—defined as witnessed arrests presenting with an initial shockable rhythm and used internationally for benchmarking outcomes—represents roughly 13 % of all OHCA cases, with survival ranging from 6 % to 27 %. Within this group, return of spontaneous circulation has been reported in 30–81 %, and survival to hospital discharge in 0–53 %, highlighting substantial variability across systems. In regions with high rates of bystander cardiopulmonary resuscitation (CPR) and structured systems for rapid defibrillation and post-arrest care, overall OHCA survival may exceed 20 % with more than 50 % survival rates in the Utstein group.^16^, ^17^

National cardiac arrest registries are a key component for improving OHCA care and research, allowing international and time-linked benchmarking, assessment of the impact of system changes, and the epidemiological changes with time. The European Resuscitation Council has prioritised the development of national registries and the study of OHCA as key components for advancing cardiac arrest care.^15^ Standardised data reporting using Utstein criteria allows international and interregional comparisons and facilitates longitudinal evaluation of survival trends. A previous national registry-based study from Qatar reported survival to hospital discharge at 8.1 % and favourable neurological outcomes at 5.4 %.^18^ This study identified important opportunities for improvement in OHCA outcomes. Qatar OHCA registry was established to enable international comparison and historical benchmark aligning with these recommendations. In this study, we aim to describe the epidemiology, management characteristics, and outcomes of OHCA within this national registry cohort.

## Methods

### Study design

This prospective observational cohort included all adult (≥18 years), non-traumatic OHCA cases in Qatar in whom resuscitation was attempted by the national EMS provider, including patients pronounced dead in the prehospital setting. Data were collected by a dedicated research team using a template designed in accordance with Utstein reporting criteria (Supplemental File 1). Data were collected directly from incident reporting and dispatch data, Emergency Medical Services (EMS) patient care records, and hospital patient Electronic Medical Records (EMR). The study involved the National EMS provider, five emergency departments, and eight hospitals. Ethical approval for this study was obtained from the Hamad Medical Corporation (HMC) in Qatar, through the Medical Research Centre Ethics Board (IRGC-07-SI-20-712).

As data collection for survivors included quality-of-life assessments, informed written consent was obtained from all survivors, or where capacity to provide consent was not regained, their next of kin, or when no next of kin was identified, from a professional legal representative. Consent and data collection were obtained in the patient’s preferred language using translated documents and professional translators. No consent was obtained for patients assessed as brain dead or those who died in the hospital, as there was no further data to be collected.

### Study setting

The state of Qatar has a unified public hospital and ambulance (Hamad Medical Corporation Ambulance Service [HMCAS]) system, HMC, with all hospitals sharing the same electronic medical record, ethical review system, and mortuary. OHCA patients are only cared for within the public system, ensuring comprehensive data collection for OHCA. The Prowar™ computerised dispatch system and HMCAS collect the Utstein reporting matrix. Paramedics collect time and physiological data using a dedicated electronic medical record. CaremonX EMR (Cerner Corporation, Kansas City, Missouri, USA) was used for hospital data collection.

For possible OHCA, HMCAS deploys a tiered system of emergency response units (ERUs): Alpha and Bravo units with two paramedics for standard care, Charlie units with a Critical Care Paramedic and assistant for advanced interventions, and Delta units led by a senior supervisor for complex cases. Helicopter retrieval is employed for patients outside the urban areas.

### Participants

The study population consisted of adult males and females aged 18 years and older who experienced non-traumatic out-of-hospital cardiac arrest (OHCA) in whom resuscitation was commenced, including those subsequently declared dead prehospital. OHCA was defined as cardiac arrest occurring outside of an inpatient hospital stay, including cases in the community, primary care settings, and assisted living facilities, provided EMS was dispatched. Accordingly, patients who experienced cardiac arrest in a primary care setting or as hospital visitors were included if resuscitation was initiated by EMS on hospital grounds, whereas cases attended exclusively by in-hospital staff were excluded.

Exclusion criteria included patients younger than 18 years, those with traumatic arrest, individuals pronounced dead upon EMS arrival with no attempts at resuscitation, patients presenting with obvious signs of death (termed as 'undeniable death' and including decapitation, incineration, decomposition, rigour mortis, and dependent lividity), and those with a Do Not Attempt Resuscitation order in place.

**Fig. 1:**
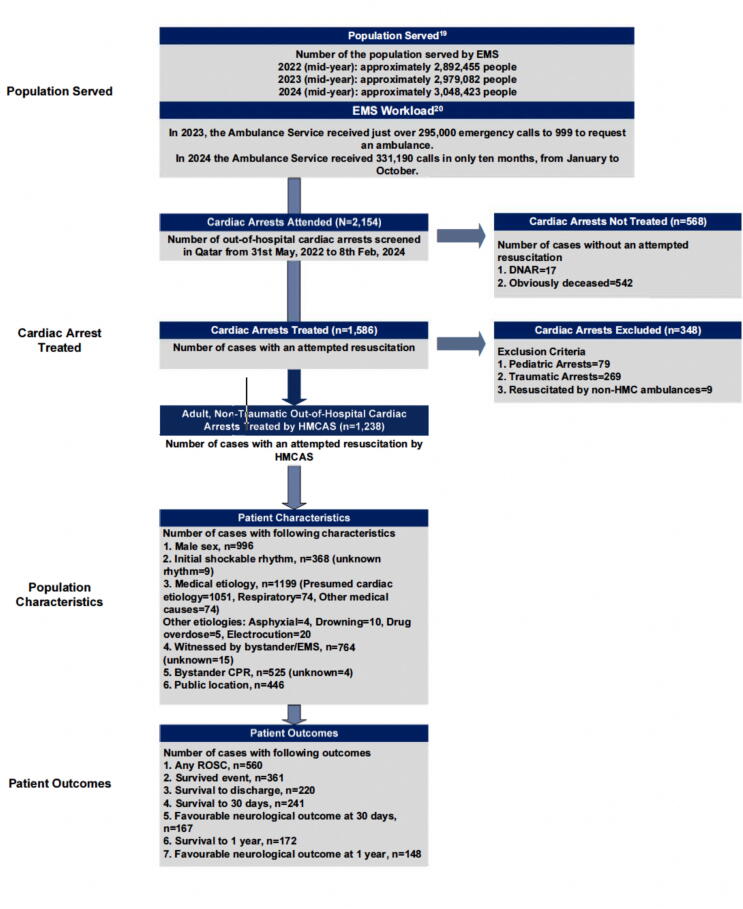
**Flow diagram of the study population. The diagram details the population served by EMS, total EMS workload, number of cardiac arrest cases screened, treated, and excluded, as well as baseline patient characteristics and key outcomes at various time points**. (19, 20)

The study flow, including the population served, EMS workload, screened cardiac arrests, inclusion and exclusion processes, baseline patient characteristics, and key outcomes, is summarised in Fig. 1.

### Data collection

Data collection was conducted by a dedicated, trained research team who were present in the Emergency Department (ED) seven days a week. The research team attended all episodes of cardiac arrest in the ED of the country’s largest hospital (which cares for >90 % OHCA patients) from 06.30 to 22.30 h, obtaining data in real time. Outside these hours and in the remaining ED’s data was collected from the EMR supplemented by direct contact with the pre- and in-hospital clinical teams The data fields were predefined by the Utstein criteria and included time points (response time, defibrillation time, transport time), aetiology of the OHCA, treatment received (bystander CPR, defibrillation, medications), and outcomes at various stages ROSC, survival to ED admission, survival to hospital discharge, 30-day survival, and neurological outcomes at 30 days).^21^

Data on patients in whom resuscitation was commenced but were declared dead in the prehospital phase were obtained from Qatar Ambulance Service records, hospital electronic medical records, and the National mortuary records. There is one mortuary for the entire country, one national ambulance service, and only the public hospitals accept patients in cardiac arrest, ensuring comprehensive and complete data collection.

Survivors were followed up in the hospital and, if discharged, at 30 days and 12 months via structured face-to-face or telephone interviews to assess CPC and performance outcomes (EQ-5D) (see supplementary material for full data collected at follow-up and the transcript template used by the research team).

### Outcomes

#### Primary outcome

The primary outcome was 30-day survival with a favourable neurological status (CPC 1 or 2) among patients for whom resuscitation was attempted following OHCA.

#### Secondary outcomes

Secondary outcomes included ROSC at any time and upon ED arrival, survival to hospital admission and hospital discharge, and rates of emergency interventions, with the associated 30-day mortality for each intervention group.

### Statistical analysis

All analyses were conducted in STATA Corp IC 16.1. Descriptive analyses for categorical variables were reported as frequencies and percentages. For continuous variables, normality was assessed visually by histogram and Q-Q plots. Means were reported for normally distributed variables, while for non-normally distributed variables, medians and Inter Quartile Ranges (IQR) were reported.

For the primary outcome of neurologically favourable survival, a comparison of the differences between the group with neurologically favourable and with neurologically unfavourable survival was made. For significance testing, categorical variables were compared using the Chi-square test and Fisher’s exact test as appropriate. Continuous variables were compared using independent *t*-test for normally distributed variables and Wilcoxon Rank Sum Test or Mann Whitney Test for non-normally distributed variables.

For the secondary outcomes, survival to event and survival to discharge, logistic regression was used to assess the associations with survival outcomes. The choice of variables was based on previous literature.^18^, ^22^ A stepwise logistic regression model was built manually using purposeful selection of variables through the backward elimination method.^18^, ^23^, ^24^ Univariate analysis was first conducted for all variables potentially associated with these outcomes. Variables with unadjusted *p*-values less than 0.2 were included in the multivariable regression model.^23^ We specified the significance of the *p*-value at 0.05 for the multivariable model. At each step, the goodness of fit of the model was assessed by the Likelihood Ratio Test and the Akaike Information Criteria (AIC) and Bayesian Information Criteria (BIC) measures. The final model contained variables that were either significant or had any confounding effect present. The goodness of fit of the model was also taken into consideration in finalising the model. Multicollinearity was assessed by the Spearman’s correlation test and moderate to highly collinear variables were removed. The model performance was assessed by the Area Under the Receiver Operator Characteristic Curve (AUROC) to assess the ability of the model to discriminate the Cardiac Arrest outcomes.

## Results

Among 1238 OHCA patients, the median age was 52 years (IQR, 41–65), and 80.5 % were male. The majority of arrests occurred at home (64.0 %), 61.8 % were witnessed, and bystander CPR was performed in 42.4 %. Initial shockable rhythms were observed in 29.7 %. Median EMS response and scene times were 7.6 and 46.8 min, respectively. Survival to hospital discharge was 17.8 % (Table 1).

**Table 1:** Baseline characteristics of Out of Hospital Cardiac Arrest (OHCA) patients.

**Baseline characteristics**	***N*, % (*N* = 1238)**
Age, median (IQR)	52.0 (41.0, 65.0) (*n* = 1238)
Male sex	996 (80.5 %)
**Ethnicity**^a^	
Qatari	203 (16.4 %)
Other Arab	266 (21.5 %)
South Asian	557 (45.0 %)
African	43 (3.5 %)
Filipino	63 (5.1 %)
Caucasian	44 (3.6 %)
Other	21 (1.7 %)
Unknown/missing	41 (3.3 %)
**Location of arrest**	
Home	792 (64.0 %)
Healthcare facility	65 (5.3 %)
Assisted living facilities	14 (1.1 %)
Public places	252 (20.4 %)
Street or highway	105 (8.5 %)
Remote areas	9 (0.7 %)
Unknown/missing	1 (0.1 %)
**Past history**	
Ability to do daily activity independently (pre-arrest)	928 (75.0 %)
Unknown/missing	78 (6.30 %)
Known history of heart disease	330 (26.7 %)
Known history of respiratory disease	144 (11.6 %)
Known history of diabetes	458 (37.0 %)
Known history of stroke	77 (6.2 %)
Known history of hyperlipidemia	212 (17.1 %)

**Arrest characteristics**	
**Arrest witnessed by**	
Not witnessed	459 (37.1 %)
Witnessed by EMS	205 (16.6 %)
Witnessed by bystanders	559 (45.2 %)
Unknown/missing	15 (1.2 %)
**First CPR performed by**	
No CPR initiated prehospital	14 (1.1 %)
EMS	695 (56.14 %)
Bystander healthcare provider	145 (11.7 %)
Bystander lay person	212 (17.1 %)
Bystander family	168 (13.6 %)
Bystander unknown type	4 (0.3 %)
Shockable initial rhythm	368 (29.7 %)
Unknown/missing	9 (0.7 %)
**First airway**	
No airway intervention done or indicated	101 (8.2 %)
Intubation	359 (29.0 %)
SGAD	763 (61.6 %)
Already tracheostomized patients at time of arrest	15 (1.2 %)
**Time intervals in median (IQR)**	
Time call to dispatch	0.00 (0.0, 1.1) (*n* = 1236)
Time call to scene	7.55 (5.6, 10.0) (*n* = 1235)
Time call to ED arrival	71.00 (60.0, 85.0) (*n* = 826)
Time EMS spent on scene (Time EMS at patient side to Time EMS left the scene)	46.85 (36.0, 59.7) (*n* = 1189)
Time to CPR by EMS	13.57 (10.3, 18.4) (*n* = 1189)
Time to AED by EMS	12.00 (9.5, 15.8) (*n* = 1232)
Time call to first shock	18.41 (12.1, 29.4) (*n* = 568)
Time call to first ROSC	32.00 (22.1, 46) (*n* = 537)
**Survival outcomes**	
Survival at 1 month	241 (19.5 %)
Survival to hospital discharge	220 (17.8 %)
Survival at 1 year	172 (13.9 %)

*Abbreviations*: EMS, Emergency Medical Services; SGAD, Supra Glottic Airway Device; IQR, Interquartile Range; ED, Emergency; CPR, Cardiopulmonary Resuscitation; AED, Automated External Defibrillator; ROSC, Return of Spontaneous Circulation

a Ethnicity is based on regions.

Patients with favourable neurological outcomes (*n* = 167) were significantly younger (47 vs. 53 years, *p* < 0.001), more likely male (93.4 % vs. 78.4 %, *p* < 0.001), had higher rates of EMS-witnessed arrests (42.5 % vs. 12.5 %, *p* < 0.001), shockable rhythms (80.8 % vs. 21.8 %, *p* < 0.001), and bystander CPR by healthcare providers. Favourable outcomes were less common following home arrests (45.5 % vs. 66.9 %, *p* < 0.001). Respiratory disease, diabetes, stroke, and amiodarone administration were associated with poor outcomes (Table 2).

**Table 2:** Characteristics and management in neurologically favourable and unfavourable out-of-hospital cardiac arrest patients in Qatar.

**Characteristics**	**Neurologically unfavourable survival** **(CPC 3 or above)** ***N* = 1071 (86.5 %)**	**Neurologically favourable survival** **(CPC 1 or 2)** ***N* = 167 (13.5 %)**	**Overall *p*-value**
Age, median (IQR)	53.0 (41.0, 66.0) (*n* = 1071)	47.0 (40.0, 55.0) (*n* = 167)	<0.001
Male sex	840 (78.4 %)	156 (93.4 %)	<0.001
Ethnicity^a^			0.48
Qatari	182 (17.0 %)	21 (12.6 %)	
Other Arab	229 (21.4 %)	37 (22.2 %)	
South Asian	475 (44.4 %)	82 (49.1 %)	
African	38 (3.6 %)	5 (3.0 %)	
Filipino	50 (4.7 %)	13 (7.8 %)	
Caucasian	37 (3.5 %)	7 (4.2 %)	
Other	19 (1.8 %)	2 (1.2 %)	
Unknown/Missing	41 (3.8 %)	0 (0.0 %)	
Location of arrest			<0.001
Home	716 (66.9 %)	76 (45.5 %)	
Healthcare facility	38 (3.6 %)	27 (16.2 %)	
Assisted living facilities	13 (1.2 %)	1 (0.60 %)	
Public places	211 (19.7 %)	41 (24.6 %)	
Street or highway	83 (7.8 %)	22 (13.2 %)	
Remote areas	9 (0.8 %)	0 (0.0 %)	
Unknown/missing	1 (0.1 %)	0 (0.0 %)	
**Past history**			
Ability to do daily activity independently (pre-arrest)	766 (71.52 %)	162 (97.01 %)	<0.001
Known history of heart disease	294 (27.5 %)	36 (21.6 %)	0.11
Known history of respiratory disease	134 (12.5 %)	10 (6.0 %)	0.01
Known history of diabetes	409 (38.2 %)	49 (29.3 %)	0.03
Known history of stroke	75 (7.0 %)	2 (1.2 %)	0.004
Known history of hyperlipidemia	191 (17.8 %)	21 (12.6 %)	0.09
**Arrest characteristics**			
Arrest witnessed by			<0.001
Not witnessed	453 (42.3 %)	6 (3.6 %)	
Witnessed by EMS	134 (12.5 %)	71 (42.5 %)	
Witnessed by bystanders	469 (43.8 %)	90 (53.9 %)	
Unknown/missing	15 (1.4 %)	0 (0.0 %)	
First CPR performed by			<0.001
No CPR initiated prehospital	1 (0.1 %)	13 (7.8 %)	
EMS	615 (57.4 %)	80 (47.9 %)	
Bystander healthcare provider	113 (10.6 %)	32 (19.2 %)	
Bystander lay person	184 (17.2 %)	28 (16.8 %)	
Bystander family	154 (14.4 %)	14 (8.4 %)	
Bystander unknown type	4 (0.4 %)	0 (0.0 %)	
Mechanical CPR			<0.001
No CPR initiated	1 (0.1 %)	12 (7.2 %)	
Mechanical CPR device	948 (88.5 %)	38 (22.8 %)	
Manual compressions	122 (11.4 %)	117 (70.1 %)	
Amiodarone	288 (26.9 %)	32 (19.2 %)	0.03
Shockable initial rhythm	233 (21.8 %)	135 (80.8 %)	<0.001
Unknown/Missing	3 (0.3 %)	6 (3.6 %)	

*Abbreviations*: EMS, Emergency Medical Services; CPR, Cardiopulmonary Resuscitation

a Ethnicity is based on regions.

Patients with favourable outcomes experienced shorter EMS intervals, including dispatch-to-scene arrival (7.0 vs. 7.7 min, *p* = 0.023), total scene duration (37.4 vs. 47.9 min, *p* < 0.001), time from dispatch to ED (67.0 vs. 72.0 min, *p* = 0.027), and time to first ROSC (22.0 vs. 34.0 min, *p* < 0.001). Coronary reperfusion within 24 h was markedly more frequent among favourable outcomes (71.3 % vs. 3.9 %, *p* < 0.001). Cardiac aetiology was more common (92.2 % vs. 83.8 %, *p* = 0.012), as was bystander Automated External Defibrillator (AED) use (11.4 % vs. 5.5 %, *p* = 0.004) (Table 3).

**Table 3:** Pre-hospital times, management and survival in neurologically favourable and unfavourable of out-of-hospital cardiac arrest patients in Qatar.

**Characteristics**	**Neurologically unfavourable survival at 1-month** **(CPC 3 or above)** ***N* = 1071 (86.5 %)**	**Neurologically favourable survival at 1-month** **(CPC 1 or 2)** ***N* = 167 (13.5 %)**	**Overall *p*-value**
**Pre-hospital times in median (IQR)**			
Time call to dispatch	0.00 (0.0, 1.1) (*n* = 1069)	0.00 (0.0, 1.1) (*n* = 167)	0.89
Time call to scene	7.74 (5.9, 10.0) (*n* = 1068)	7.00 (5.0, 9.1) (*n* = 167)	0.02
Time call to ED arrival	72.00 (60.9, 85.0) (*n* = 660)	67.00 (55.2, 83.9) (*n* = 166)	0.03
Time EMS spent on scene^a^	47.92 (38.0, 61.0) (*n* = 1024)	37.37 (26.0, 51.2) (*n* = 165)	<0.001
Time to CPR by EMS	13.43 (10.3, 18.3) (*n* = 1052)	14.10 (10.0, 26.4) (*n* = 137)	0.12
Time to AED by EMS	12.08 (9.6, 16.0) (*n* = 1065)	11.88 (9.0, 14.7) (*n* = 167)	0.09
Time call to first shock	19.00 (12.4, 28.1) (*n* = 439)	16.33 (10.9, 36.8) (*n* = 129)	0.35
Time call to first ROSC	34.00 (25.8, 51.5) (*n* = 380)	22.80 (16.0, 38.0) (*n* = 157)	<0.001
**Cause of arrest**			0.01
Presumed cardiac aetiology	897 (83.8 %)	154 (92.2 %)	
Respiratory	75 (7.0 %)	3 (1.8 %)	
Electrocution	20 (1.9 %)	0 (0.0 %)	
Drowning	9 (0.8 %)	1 (0.6 %)	
Drug overdose	3 (0.3 %)	2 (1.2 %)	
Other causes	67 (6.3 %)	7 (4.2 %)	
**Coronary reperfusion within 24 h**			<0.001
No reperfusion indicated	1030 (96.2 %)	48 (28.7 %)	
Thrombolysis	19 (1.8 %)	2 (1.2 %)	
Coronary Angiography (CAG)	4 (0.4 %)	13 (7.8 %)	
Percutaneous Coronary Intervention (PCI)	18 (1.7 %)	103 (61.7 %)	
Coronary Artery Bypass Grafting (CABG)	0 (0.0 %)	1 (0.6 %)	
Survival at 1 month	74 (6.9 %)	167 (100.0 %)	<0.001
Survival to hospital discharge	53 (4.9 %)	167 (100.0 %)	<0.001
Dispatcher-assisted CPR (DA-CPR)	186 (17.4 %)	27 (16.2 %)	0.70
Bystander AED	59 (5.5 %)	19 (11.4 %)	0.004
Bystander shockable witnessed (Utstein comparator group)	137 (12.8 %)	67 (40.1 %)	<0.001

*Abbreviations*: IQR, Interquartile Range; ED, Emergency Department; CPR, Cardiopulmonary Resuscitation; EMS, Emergency Medical Services; AED, Automated External Defibrillator; ROSC, Return of Spontaneous Circulation

a Time from EMS arrival at patient side to Time EMS left the scene.

Patients meeting the Utstein criteria (bystander-witnessed arrests with initial shockable rhythm) had significantly higher prehospital ROSC (58.3 % vs. 37.8 %) and survival to hospital discharge (38.2 % vs. 13.7 %; both *p* < 0.001) (Table 4).

**Table 4:** Clinical outcomes of OHCA patients stratified by initial shockable versus non-shockable rhythm and the Utstein comparator group (bystander-witnessed arrests with initial shockable rhythm) among OHCA patients.

**Characteristics**	**First rhythm shockable**	**First rhythm non-shockable**	***p*-value**	**Shockable bystander witnessed**	**Not shockable bystander witnessed**	***p*-value**
	**(*n* = 368)**	**(*n* = 861)**		**(*n* = 204)**	**(*n* = 1034)**	
Any ROSC (pre-hospital or ED)	249 (67.6 %)	302 (35.0 %)	<0.001	134 (65.6 %)	426 (41.2 %)	<0.001
ROSC pre-hospital	230 (62.5 %)	271 (31.4 %)	<0.001	119 (58.3 %)	391 (37.8 %)	<0.001
Any ROSC in ED	218 (59.2 %)	242 (28.1 %)	<0.001	119 (58.3 %)	350 (33.8 %)	0.009
Survival at 1 month	156 (42.3 %)	76 (8.8 %)	<0.001	80 (39.2 %)	161 (15.5 %)	<0.001
Survival to hospital discharge	152 (41.3 %)	59 (6.8 %)	<0.001	78 (38.2 %)	142 (13.7 %)	<0.001
Survival at 1 year	127 (34.5 %)	39 (4.5 %)	<0.001	65 (31.9 %)	107 (10.3 %)	<0.001

On multivariable analysis, witnessed arrest, prehospital defibrillation, and respiratory disease independently predicted ROSC, whereas mechanical chest compression and higher adrenaline dose were negatively associated (Supplemental Table S1). Survival to discharge was associated with witnessed arrest, initial shockable rhythm, and early coronary reperfusion, with mechanical compression and adrenaline dose again negatively associated (Supplemental Table S2). Supplemental Fig. S2 shows the coefficient plots from the final multivariable logistic regression analyses for ROSC at ED presentation and survival to hospital discharge. Discriminative performance was excellent (AUC 0.92 for ROSC; 0.94 for survival, Supplemental Figs. S1 and S2). At 1 year, most survivors demonstrated favourable neurological and functional recovery (CPC 1–2/Overall Performance Category 1), with EQ-5D indicating preserved or improved health status (Supplemental Tables S3–S5).

## Discussion

The findings of this study indicate marked improvements in OHCA outcomes in Qatar. Data presented here show a 30-day favourable neurological outcome of 13.5 %, survival to hospital discharge of 17.8 %, and ROSC of 44.8 %. These outcomes represent more than a two-fold improvement compared to previously reported national data from 2012 to 2013, which reported a favourable neurological outcome of 5.4 %, survival to discharge of 8.1 %, and ROSC of 13.0 %.^18^ At a global level, these results add contemporary, population-based OHCA data from a rapidly developing health system and illustrate how survival and neurological outcomes evolve when national EMS processes, documentation, and post-arrest pathways mature, while also contributing structured follow-up information that remains limited in many international registries. Several factors likely contributed to these gains, including reductions in EMS response intervals, a higher proportion of initial shockable rhythms, improved case ascertainment through active screening of all EMS cardiac arrest reports, and system-level changes such as the introduction of an OHCA-specific Cerner form, expanded multisite collaboration, and double verification of bystander CPR. Importantly, these system-level upgrades also produced a more complete and structured data collection, which may partly explain the apparent improvement in reported outcomes compared with earlier estimates. At the same time, progressive enhancements in prehospital and in-hospital cardiac arrest care—such as more consistent early defibrillation, refined post-ROSC pathways, and greater integration between EMS and receiving hospitals—likely contributed to genuine clinical gains. Thus, the improved results likely reflect a combination of better case capture and meaningful advances in the quality of resuscitation and post-arrest care across the system. These figures also compare favourably with international studies, where 30-day survival is reported at approximately 10.7 %, the rate of survival to discharge is around 8.8 %, and ROSC rates are close to 29.7 %.^23^ The observed differences may reflect a combination of system-level efficiencies and population-specific factors influencing resuscitation success and post-arrest care. In contrast, OHCA outcomes reported across Gulf countries remain limited. ROSC rates in the region demonstrate wide variability, with estimates ranging from 1.6 % in Kuwait to 39.4 % in a single-centre study in Oman.^24^, ^25^ A scoping review of OHCA in the Gulf Cooperation Council (GCC) states described survival to hospital discharge ranging from 1 % to 13 %.^26^ In Saudi Arabia, the national OHCA registry documented a hospital discharge survival rate of 2.9 %, with favourable neurological outcomes (CPC 1–2) in less than 0.5 % of cases.^27^ In Bahrain, the corresponding survival rate was 1.2 %, with no reported cases of good neurological recovery.^28^

The demographic profile of our study population—predominantly younger adults with a mean age of 52 years—differs markedly from international OHCA cohorts. In Europe, the EuReCa TWO study reported a median OHCA age of 67.6 years, while the CARES registry in the United States documented a median of 65 years.^2^, ^29^ In the Aus-ROC Epistry, the median age was 68 years in Australia and 66 years in New Zealand.^30^ Across seven countries in Asia, the Pan-Asian Resuscitation Outcomes Study (PAROS) reported median ages ranging from 49.7 to 71.7 years, depending on local population demographics.^31^

Within the Gulf region, including Qatar, reported OHCA cohorts tend to be substantially younger. Reported estimates from GCC countries indicate median ages ranging from 49 to 62.9 years, consistent with regional demographic patterns.^26^ This pattern is largely attributable to the distinct male labourer-dominated population structures in the Gulf. In Qatar, expatriates comprise approximately 89 % of the total population, and about 75 % of the non-citizen workforce are males.^32^, ^33^ As a result, regional OHCA registries, including ours, capture a predominantly young and male cohort. This younger age profile may offer clinical advantages, including greater physiological reserve, which can enhance survival and neurological outcomes. Although female sex was not an independent predictor of survival in our multivariable model, interpretation of sex-specific differences is limited by the small number of female survivors with CPC 1–2 (6.6 %). A recent national gender-based analysis from Qatar similarly showed that females experiencing OHCA tend to be older, arrest less often in public settings, and present with shockable rhythms less frequently—factors associated with lower survival probabilities.^34^ These contextual patterns suggest that the apparent survival advantage among younger males in our cohort is more likely related to arrest characteristics and baseline risk profiles rather than intrinsic physiological differences between sexes.

The median EMS dispatch-to-scene arrival interval was 7.5 min, representing a decrease from the 8.7-min interval reported in Qatar in 2016. This response interval aligns with international benchmarks, including 7–9 min in the United States (CARES), 8.4 min in Australia (Aus-ROC), and 8–10 min in European settings (EuReCa TWO), and is comparable to estimates from other Gulf countries such as Saudi Arabia (10 min) and Bahrain (9 min).^2^, ^27^, ^28^, ^29^, ^30^ The proportion of initial shockable rhythms was 29.7 %, aligning with international estimates from the CARES registry (28.8 %) and the EuReCa TWO study (20.2 %), and exceeding rates reported in regional datasets, including Saudi Arabia (10.4 %) and the United Arab Emirates(UAE) (12.5 %).^2^, ^27^, ^29^, ^30^, ^35^ This relatively high prevalence may partially account for the observed survival and neurological outcome rates, given the established prognostic significance of shockable rhythms in OHCA. Shockable rhythms contributed disproportionately to favourable outcomes, with 80.8 % of CPC 1–2 survivors presenting in this category. This concentration of survivorship within the shockable cohort suggests that the overall improvement in outcomes may have been driven not only by system-level changes but also by a higher likelihood of identifying and treating these time-sensitive rhythms before irreversible physiology develops. In contrast, outcomes among patients with non-shockable rhythms remained poor, in keeping with the limited reversibility of PEA and asystole and their weaker association with survival in our adjusted analysis. Several structural features observed in this setting—such as high population density, short EMS response intervals, bystander CPR provision, and coordinated prehospital-hospital care pathways—are consistent with characteristics reported in high-performing systems like Seattle, where CPC 1–2 survival following OHCA approaches 36 %.^36^ These system-level attributes may contribute to the observed rates of favourable neurological outcomes.

Scene times in this cohort were prolonged, with a median duration of 46.8 min—markedly longer than reported in the United States (15–20 min, CARES registry) and Europe (18–25 min, EuReCa TWO).^2^, ^31^ Similar findings were described in a 2016 national study from Qatar, which reported a median scene time of 37.9 min.^18^ This reflects a service model in which EMS-treated cardiac arrests are generally attended by a critical care paramedic, allowing comprehensive advanced life support interventions to be delivered on scene. Although extracorporeal support is not provided prehospital, all other ALS modalities—including airway management, defibrillation, drug administration, and vascular access—are routinely available. Field termination-of-resuscitation is seldom applied, as previously reported in Qatar, and resuscitative efforts typically continue unless unequivocal signs of death are present.^18^ In contrast, many western EMS systems employ validated TOR rules that allow earlier cessation of futile resuscitation attempts, resulting in shorter scene times in those contexts.^37^ Several studies have reported that transporting patients while resuscitation is ongoing may be associated with lower survival, particularly when ROSC has not been achieved before movement from the scene.^38^, ^39^ The 2022 International Consensus on CPR and ECC similarly suggests providing resuscitation at the scene rather than initiating transport during ongoing CPR, unless transport is required to access an intervention such as extracorporeal membrane oxygenation.^40^ Although these data do not establish a single optimal operational strategy, they indicate that extended on-scene resuscitation—when full ALS capability is available—can remain compatible with contemporary guideline recommendations and may support the likelihood of achieving ROSC before transport.

The rate of bystander CPR in this cohort was 42.4 %, comparable to international estimates reported in the United States (CARES: ∼40 %) and lower than those observed in Europe (EuReCa TWO: 58 %).^2^, ^29^ This rate exceeds figures reported in regional studies from Saudi Arabia (27.7 %) and the UAE (30.7 %).^27^, ^35^ While health-care and EMS systems across Gulf countries are not identical, comparisons still provide useful regional context. Variations in EMS structure, dispatch models, and demographic composition—such as differences in age distribution, comorbidities, and expatriate workforce proportions—may influence observed OHCA patterns. The comparisons in our study were therefore intended to situate the findings within the regional landscape rather than to imply direct equivalence between health systems. Bystander AED use was documented in 6.3 % of cases, which is within the global range (2–12.8 %) and higher than previously reported in Qatar (2.7 % in 2016), as well as rates from Saudi Arabia (2.5 %) and the UAE (1.8 %).^18^, ^27^, ^35^, ^41^ Given that bystander CPR and AED use substantially increase the likelihood of early defibrillation and survival, these improvements likely contributed—at least in part—to the higher ROSC and survival rates observed in this cohort. Shortening the time to first chest compression is particularly important in Qatar, where a large proportion of arrests occur at home and EMS response intervals, although improving, remain longer than in some high-performing systems. Despite this encouraging trend, there remains substantial room for further improvement. Language diversity and the high turnover among the expatriate population continue to pose challenges to sustained AED education and engagement. Targeted interventions—such as multilingual CPR/AED training, digital platforms for AED location, and culturally tailored public outreach—may help address these barriers and further strengthen early bystander response.

Among patients who met the Utstein comparator definition (bystander-witnessed cardiac arrests with an initial shockable rhythm), the survival to hospital discharge in our cohort was 38.2 %, with ROSC at hospital arrival observed in 58.8 %, and CPC 1–2 neurological outcome achieved in 32.8 % of cases. These figures are broadly aligned with outcomes reported in large-scale OHCA registries from North America, Europe, and Australasia. In the EuReCa TWO study, survival and ROSC rates were 28.0 % and 59.0 %, respectively.^2^ The 2023 CARES registry documented 36.3 % survival, 50.2 % sustained ROSC, and 28.9 % CPC 1–2 outcome among Utstein cases.^29^ Australian data from the Aus-ROC Epistry reported a survival rate of 34.2 % and ROSC at hospital arrival in 49.9 % of cases, while in New Zealand, the corresponding rates were 29.2 % and 48.7 %.^30^

In this cohort, 64 % of OHCAs occurred in residential settings, consistent with international estimates. Home-based arrests account for the majority of events across multiple registries, including 71 % in the United States (CARES), 70.2 % in Europe (EuReCa TWO), 65.4 % in Asia (PAROS), and 76 % in Australia (Aus-ROC Epistry).^2^, ^29^, ^30^, ^31^ Reports from GCC countries indicate a broader range (54–85 %), reflecting regional variability in EMS accessibility and population structure.^26^ Residential arrests are generally associated with prolonged recognition intervals, reduced bystander intervention rates, and lower survival probability compared to public or occupational settings.

In multivariable analyses, witnessed arrest and prehospital defibrillation were the strongest predictors of ROSC at ED arrival, while mechanical chest compression and multiple doses of adrenaline were independently associated with reduced likelihood of ROSC. Survival to hospital discharge was positively associated with witnessed arrest, initial shockable rhythm, and early coronary reperfusion. These findings underscore the importance of early recognition, prompt defibrillation, and coordinated post-resuscitation care in improving OHCA outcomes.

### Limitations

This study has limitations that merit consideration. The sample size was modest compared with larger international registries; however, data quality was high, with real-time prospective data collection by trained personnel rather than retrospective EMR abstraction. Although conducted within a single national system, the integrated structure of Qatar’s EMS, emergency hospitals, and mortuary services ensured near-complete case ascertainment, strengthening internal validity. Compared with the 2015 nationwide OHCA study,^18^ the current registry further improved ascertainment through attempted real-time ED data capture, expanded multisite coverage, and mortuary record integration, thereby reducing data loss and enhancing the reliability and generalizability of the findings. Nonetheless, some sources of bias may persist. Research assistants were not physically present for every OHCA admission, thus real-time verification was not always feasible. Comorbidities were recorded as binary variables, which may not fully reflect heterogeneity within disease groups and could contribute to residual confounding in adjusted analyses. Loss to follow-up occurred in a minority of patients by 12 months, yet structured assessments of functional and health-related quality of life (EQ-5D and visual analogue/numeric rating scales) were obtained in most survivors, providing a depth of outcome characterization seldom reported in comparable cohorts. Baseline pre-arrest functional status was also systematically recorded, with the majority of patients independent in activities of daily living—an infrequently captured but clinically critical variable for interpreting outcomes. Despite these constraints, the prospective design, multisite enrollment, and linkage across EMS, ED, and mortuary data substantially mitigate the risk of missed eligible cases and support the overall internal validity of the findings.

## Conclusions

This national OHCA registry demonstrates a more than two-fold improvement in survival and CPC 1–2 neurological outcomes compared with prior Qatari data. Absolute survival rates now align with those reported from countries with mature resuscitation systems. Known predictors of favourable outcome (witnessed arrest, early defibrillation, and timely coronary reperfusion) were consistent with earlier published studies, reinforcing their prognostic value in this setting.

## Consent for publication

Not Applicable.

## Availability of data and materials

The datasets used and/or analysed during the current study are available from the corresponding author on reasonable request.

## Clinical trial number

Not Applicable.

## CRediT authorship contribution statement

**Yavuz Yigit:** Writing – review & editing, Writing – original draft, Supervision, Project administration, Formal analysis, Data curation. **Peter Alistair Cameron:** Writing – review & editing, Writing – original draft, Supervision, Project administration. **Jassim Al Suwaidi:** Writing – review & editing, Project administration, Methodology. **Loua Al Shaikh:** Writing – review & editing, Project administration, Methodology. **Ibrahim Fawzy Hassan:** Writing – review & editing, Project administration, Methodology. **Nidal Asaad:** Writing – review & editing, Project administration, Methodology. **Nicholas Castle:** Writing – review & editing, Project administration, Methodology. **Ian Lucas Howard:** Project administration, Methodology, Data curation. **Abdulrahman Arabi:** Writing – review & editing, Project administration, Methodology. **Atika Jabeen:** Writing – review & editing, Formal analysis, Data curation. **Tim Richard Edmund Harris:** Writing – review & editing, Writing – original draft, Supervision, Project administration, Methodology, Funding acquisition, Conceptualization.

## Ethics approval and consent to participate

Ethical approval for this study was obtained from the Hamad Medical Corporation (HMC) in Qatar, through the Medical Research Centre Ethics Board (IRGC-07-SI-20-712). The study was conducted in full conformance with principles of the “Declaration of Helsinki”, Good Clinical Practise (GCP) and within the laws and regulations of Ministry of Public Health in Qatar. Informed written consent was obtained from all survivors included in the study. For patients who did not regain capacity to provide consent, consent was obtained from their next of kin. All consent procedures were conducted in the patient’s preferred language using professionally translated documents and certified interpreters.

## Funding

The trail was funded by Medical Research Centre at 10.13039/100007833Hamad Medical Corporation, Doha, Qatar (IRGC-07-SI-20-712).

## Declaration of competing interest

The authors declare the following financial interests/personal relationships which may be considered as potential competing interests: Atika Jabeen reports financial support was provided by Hamad Medical Corporation. Atika Jabeen reports a relationship with Hamad Medical Corporation that includes: employment and funding grants. If there are other authors, they declare that they have no known competing financial interests or personal relationships that could have appeared to influence the work reported in this paper.
