# Supplementary material for: Out-of-hospital cardiac arrest in Qatar: epidemiology, management, and outcomes from a national registry study
Source: Resusc Plus. 2025 Dec 22;28:101200. doi: 10.1016/j.resplu.2025.101200 (PMC12906069; doi:10.1016/j.resplu.2025.101200)
Supplement: Supplementary Figures [file mmc1.docx]

**Supplemental Figure S1:** Coefficient Plots of the association of the predictors of Out-of-Hospital Cardiac Arrest Outcomes

A: Coefficient Plot of ROSC at ED Presentation

B: Coefficient Plot of Survival to Hospital Discharge


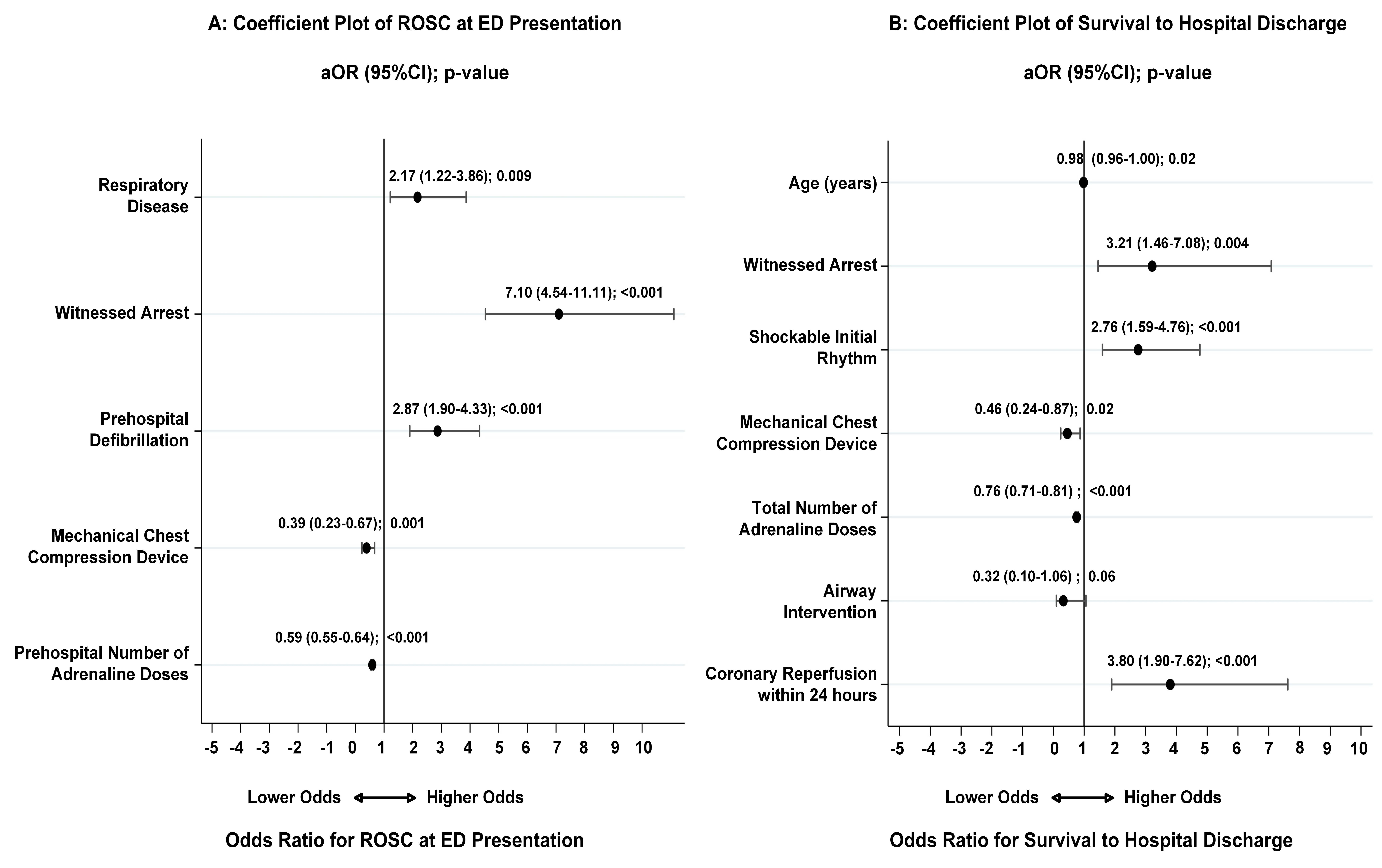


Abbreviations: ROSC, Return of Spontaneous Circulation; ED, Emergency Department

**Supplemental Figure S2:** Receiver Operating Characteristics (ROC) curves for Out-of-Hospital Cardiac Arrest Outcomes

A: ROC Curve for ROSC at ED Presentation

B: ROC Curve for Survival to Hospital Discharge


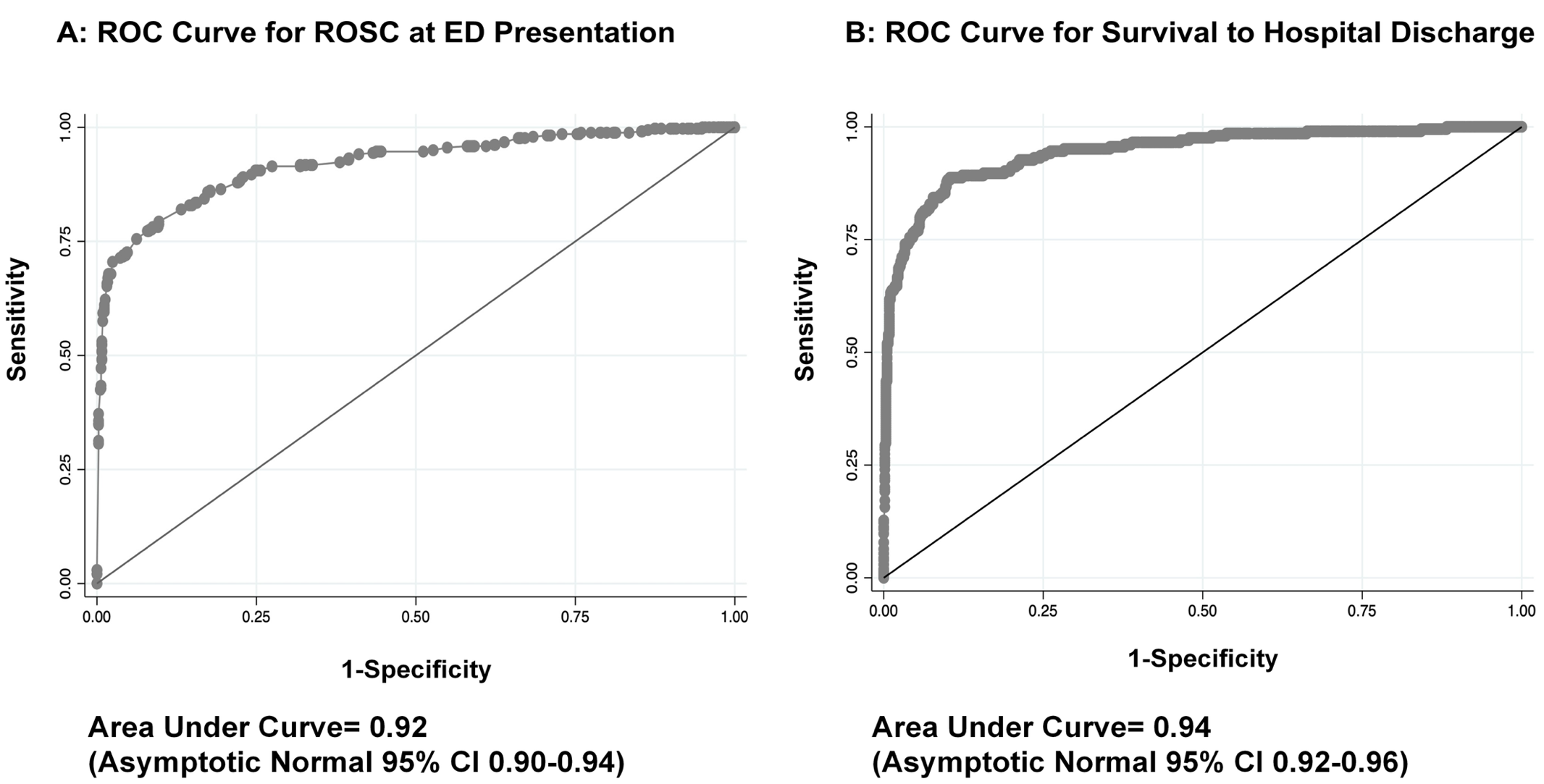


Abbreviations: ROSC, Return of Spontaneous Circulation; ED, Emergency Department
