# Supplementary material for: Out-of-hospital cardiac arrest in Qatar: epidemiology, management, and outcomes from a national registry study
Source: Resusc Plus. 2025 Dec 22;28:101200. doi: 10.1016/j.resplu.2025.101200 (PMC12906069; doi:10.1016/j.resplu.2025.101200)
Supplement: Supplementary Tables [file mmc2.docx]

**Supplemental Table S1:** Predictors of Out-of-Hospital Cardiac Arrest Outcomes

| Prehospital Variables | Relative Odds of ROSC at ED Presentation | | | | | |
| --- | --- | --- | --- | --- | --- | --- |
|  | Unadjusted Odds Ratios | p-value | 95% CI | Adjusted Odds Ratios | p-value | 95% CI |
| Age (in years) | 1.00 | 0.88 | 0.99-1.01 | . | . | . |
| Male | 1.08 | 0.63 | 0.78-1.50 | . | . | . |
| Ethnicity^1^ (Qatar as comparator) |  |  |  | . | . | . |
| Other Arabs | 0.98 | 0.92 | 0.65-1.47 | . | . | . |
| South Asians | 0.75 | 0.12 | 0.52-1.08 | . | . | . |
| Africans | 0.66 | 0.30 | 0.30-1.44 | . | . | . |
| Filipino | 1.23 | 0.50 | 0.67-2.25 | . | . | . |
| Caucasians | 1.10 | 0.79 | 0.55-2.22 | . | . | . |
| Others | 0.79 | 0.65 | 0.29-2.15 | . | . | . |
| Location of Arrest: Home | 0.89 | 0.37 | 0.68-1.15 | . | . | . |
| Heart Disease | 1.13 | 0.40 | 0.85-1.50 | . | . | . |
| Hypertension | 1.06 | 0.68 | 0.82-1.37 | . | . | . |
| Respiratory Disease | 1.32 | 0.16 | 0.90-1.94 | 2.17 | 0.009 | 1.22-3.86 |
| Diabetes | 0.95 | 0.72 | 0.73-1.24 | . | . | . |
| Stroke | 1.15 | 0.60 | 0.68-1.93 | . | . | . |
| Hyperlipidemia | 1.14 | 0.44 | 0.82-1.58 | . | . | . |
| Witnessed arrest | 6.19 | <0.001 | 4.33-8.83 | 7.10 | <0.001 | 4.54-11.11 |
| Bystander CPR | 0.92 | 0.53 | 0.71-1.19 | . | . | . |
| Shockable Initial Rhythm | 3.72 | <0.001 | 2.83-4.87 | . | . | . |
| Prehospital Defibrillation | 2.00 | <0.001 | 1.54-2.59 | 2.87 | <0.001 | 1.90-4.33 |
| Mechanical Chest Compression Device | 0.07 | <0.001 | 0.05-0.10 | 0.39 | 0.001 | 0.23-0.67 |
| Prehospital Number of Adrenaline Doses | 0.59 | <0.001 | 0.55-0.63 | 0.59 | <0.001 | 0.55-0.64 |
| Prehospital Airway Intervention | 0.03 | <0.001 | 0.01-0.06 | . | . | . |
| Scene Time^2^ | 0.99 | 0.27 | 0.97-1.01 | . | . | . |
| Time EMS spent on scene^3^ | 0.99 | 0.02 | 0.99-1.00 | . | . | . |

Abbreviations: ROSC, Return of Spontaneous Circulation; ED, Emergency Department; OHCA, Out-of-Hospital Cardiac Arrest; CPR, Cardiopulmonary Resuscitation; EMS, Emergency Medical Services

^1^Ethnicity is based on regions

^2^Scene Time=Time duration from the Time of call to the Time EMS arrived on the scene

^3^Time EMS spent on scene=Time duration from Time EMS arrived at Patient side to Time Left the Scene

**Supplemental Table S2:** Predictors of Out-of-Hospital Cardiac Arrest Survival to Hospital Discharge

| Prehospital and In-Hospital Variables | Survival to Hospital Discharge (n=774) | | | | | |
| --- | --- | --- | --- | --- | --- | --- |
|  | **Unadjusted Odds Ratios** | **p-value** | **95% CI** | **Adjusted Odds Ratios** | **p-value** | **95% CI** |
| Age (in years) | 0.98 | <0.001 | 0.97-0.99 | 0.98 | 0.02 | 0.96-1.00 |
| Male | 2.09 | 0.002 | 1.30-3.34 | . | . | . |
| Ethnicity^1^ (Qatar as comparator) |  |  |  | . | . | . |
| Other Arabs | 1.09 | 0.74 | 0.66-1.81 | . | . | . |
| South Asians | 1.11 | 0.64 | 0.71-1.73 | . | . | . |
| Africans | 1.13 | 0.79 | 0.46-2.77 | . | . | . |
| Filipino | 1.34 | 0.43 | 0.65-2.79 | . | . | . |
| Caucasians | 0.63 | 0.31 | 0.26-1.55 | . | . | . |
| Others | 0.74 | 0.66 | 0.20-2.78 | . | . | . |
| Location of Arrest: Home | 0.56 | <0.001 | 0.40-0.77 | . | . | . |
| Heart Disease | 0.80 | 0.22 | 0.55-1.15 | . | . | . |
| Hypertension | 0.74 | 0.07 | 0.53-1.02 | . | . | . |
| Respiratory Disease | 0.51 | 0.02 | 0.29-0.90 | . | . | . |
| Diabetes | 0.76 | 0.11 | 0.54-1.06 | . | . | . |
| Stroke | 0.46 | 0.09 | 0.19-1.12 | . | . | . |
| Hyperlipidemia | 0.75 | 0.18 | 0.49-1.14 | . | . | . |
| Witnessed arrest | 8.52 | <0.001 | 4.41-16.49 | 3.21 | 0.004 | 1.46-7.08 |
| Bystander CPR | 1.11 | 0.54 | 0.80-1.53 | . | . | . |
| Shockable Initial Rhythm | 7.17 | <0.001 | 4.99-10.32 | 2.76 | <0.001 | 1.59-4.76 |
| Defibrillation | 2.88 | <0.001 | 1.96-4.25 | . | . | . |
| Mechanical Chest Compression Device | 0.05 | <0.001 | 0.04-0.08 | 0.46 | 0.02 | 0.24-0.87 |
| Total Number of Adrenaline Doses | 0.66 | <0.001 | 0.62-0.70 | 0.76 | <0.001 | 0.71-0.81 |
| Airway Intervention | 0.01 | <0.001 | 0.004-0.03 | 0.32 | 0.06 | 0.10-1.06 |
| Scene Time^2^ | 0.99 | 0.56 | 0.96-1.02 | . | . | . |
| Transport Time^3^ | 1.00 | 0.38 | 0.99-1.00 | . | . | . |
| Time EMS spent on scene^4^ | 0.99 | 0.02 | 0.98-1.00 | . | . | . |
| Coronary reperfusion within 24 hours | 20.65 | <0.001 | 13.05-32.69 | 3.80 | <0.001 | 1.90-7.62 |

Abbreviations: CPR, CardioPulmonary Resuscitation; EMS, Emergency Medical Services

^1^Ethnicity is based on regions

^2^Scene Time=Time duration from the Time of call to the Time EMS arrived on the scene

^3^Transport Time=Time duration from the Time of call at the Patient side to the Time EMS arrived at ED

^4^Time EMS spent on scene=Time duration from Time EMS arrived at Patient side to Time Left the Scene

**Supplemental Table S3:** Neurologically favorable and unfavorable survival and CPC/OPC Categories among 1-month and 1-year Survivors

| **CPC/OPC Categories with Description** | **Frequency** | **Percentage** |
| --- | --- | --- |
| **1-month Survivors** | **241** | **100.0%** |
| **Neurologically Favorable Survival** | **167** | **69.3%** |
| 1-Good cerebral/overall performance | 157 | 65.1% |
| 2-Moderate cerebral/overall disability | 10 | 4.1% |
| **Neurologically Unfavorable Survival** | **74** | **30.7%** |
| 3-Severe cerebral/overall disability | 10 | 4.1% |
| 4-Coma or vegetative state | 63 | 26.1% |
| 5-Confirmed Brain Dead | 1 | 0.4% |
| **1-year Survivors** | **172** | **100.0%** |
| **Neurologically Favorable Survival** | **148** | **86.0%** |
| 1-Good cerebral/overall performance | 144 | 83.7% |
| 2-Moderate cerebral/overall disability | 4 | 2.3% |
| **Neurologically Unfavorable Survival** | **24** | **14.0%** |
| 3-Severe cerebral/overall disability | 10 | 5.8% |
| 4-Coma or vegetative state | 14 | 8.1% |
| 5-Confirmed Brain Dead | 0 | 0% |

**Supplemental Table S4:** Healthcare Quality of life by the EuroQuol 5-dimensions among OHCA survivors at 1 month and 1 year

| **EQ-5D^1^** | **Description** | **1 month** | **1 year** |
| --- | --- | --- | --- |
| Mobility^1^ | Confined to bed | 72 | 24 |
|  | No problem in mobility | 152 | 141 |
|  | Some problems in mobility | 17 | 7 |
|  | **Totals** | **241** | **172** |
| Self care^1^ | Unable to wash or dress | 74 | 24 |
|  | No problem in self care | 154 | 143 |
|  | Some problems in self care | 13 | 5 |
|  | **Totals** | **241** | **172** |
| Ability to perform usual activities^1^ | Unable to perform usual activites | 74 | 24 |
|  | No problem in performing usual activites | 147 | 143 |
|  | Some problems in performing usual activites | 20 | 5 |
|  | **Totals** | **241** | **172** |
| Pain or discomfort^2^ | No pain or discomfort | 91 | 129 |
|  | Moderate pain or discomfort | 61 | 13 |
|  | Extreme pain or discomfort | 1 | 0 |
|  | Totals | 153 | 142 |
| Anxiety or depression^2^ | No anxiety or depression | 116 | 129 |
|  | Moderate anxiety or depression | 36 | 11 |
|  | Extreme anxiety or depression | 1 | 2 |
|  | **Totals** | **153** | **142** |

Abbreviations: EQ5D, EuroQuol 5-dimensions

^1^ The Assessment of the Patient’s Health and Quality of life by the EuroQuol 5-dimensions for mobility, self-care and ability to perform usual activities was done by face-to-face follow up, Phone call follow up, a combination of face-to-face and phone call follow up, or information extracted from notes in Electronic Medical Records (EMR) as applicable.

^2^ The Assessment of pain/discomfort and anxiety/depression was only done by face-to-face follow up, Phone call follow up, or a combination of face-to-face and phone call follow up.

**Supplemental Table S5**: Patient-reported Health State on Visual Analogue Scale/ Numeric Rating Scale from 0-100 and Cerebral Performance and Overall Performance Scores of OHCA survivors at 1 month and 1 year

|  | CPC/OPC Scores^1^ at 1 month | | |  |
| --- | --- | --- | --- | --- |
| Patient-reported VAS/NRS^2^ at 1 month | 1 | 2 | 3 | Total |
| 10 | 2 | 0 | 0 | 2 (1.3%) |
| 20 | 1 | 0 | 0 | 1 (0.7%) |
| 30 | 3 | 0 | 0 | 3 (2.0%) |
| 40 | 2 | 1 | 0 | 3 (2.0%) |
| 50 | 7 | 3 | 0 | 10 (6.7%) |
| 60 | 7 | 2 | 1 | 10 (6.7%) |
| 65 | 4 | 0 | 0 | 4 (2.7%) |
| 70 | 23 | 1 | 0 | 24 (16.0%) |
| 75 | 10 | 1 | 0 | 11 (7.3%) |
| 80 | 34 | 0 | 0 | 34 (22.7%) |
| 85 | 3 | 0 | 0 | 3 (2.0%) |
| 90 | 33 | 0 | 0 | 33 (22.0%) |
| 95 | 1 | 0 | 0 | 1 (0.7%) |
| 99 | 1 | 0 | 0 | 1 (0.7%) |
| 100 | 10 | 0 | 0 | 10 (6.7%) |
| Total | 141 | 8 | 1 | 150 |
|  | CPC/OPC Scores^1^ at 1 year | | |  |
| Patient-reported VAS/NRS^2^ at 1 year | 1 | 2 | 3 | Total |
| 50 | 1 | 0 | 1 | 2 (1.4%) |
| 60 | 0 | 2 | 0 | 2 (1.4%) |
| 65 | 0 | 1 | 0 | 1 (0.7%) |
| 70 | 5 | 0 | 0 | 5 (3.6%) |
| 75 | 1 | 0 | 0 | 1 (0.7%) |
| 80 | 13 | 0 | 0 | 13 (9.4%) |
| 85 | 8 | 0 | 0 | 8 (5.8%) |
| 90 | 25 | 0 | 0 | 25 (18.0%) |
| 95 | 16 | 0 | 0 | 16 (11.5%) |
| 98 | 2 | 0 | 0 | 2 (1.4%) |
| 99 | 5 | 0 | 0 | 5 (3.6%) |
| 100 | 59 | 0 | 0 | 59 (42.4%) |
| Total | 135 | 3 | 1 | 139 |

Abbreviations: CPC, Cerebral Performance Score; OPC, Overall Performance Score; VAS, Visual Analogue Scale; NRS, Numeric Rating Scale

^1^The Assessment of the Cerebral Performance and Overall Performance Scores was done by face-to-face follow up, Phone call follow up, or a combination of face-to-face and phone call follow up as applicable.

^2^The Visual Analogue Scale (VAS) comprised a scale from 0-100, with 0 considered as the worst imaginable health state and 100 as the best imaginable health state. Depending upon the type of follow up (face-to-face, or phone call), either the VAS or the Numeric Rating Scale (NRS) was utilised. The patient-reported health states were only applicable to cases with CPC/OPC 3 and below.
