# Supplementary material for: Out-of-hospital cardiac arrest in Qatar: epidemiology, management, and outcomes from a national registry study
Source: Resusc Plus. 2025 Dec 22;28:101200. doi: 10.1016/j.resplu.2025.101200 (PMC12906069; doi:10.1016/j.resplu.2025.101200)
Supplement: Supplementary File 1 [file mmc3.pdf]

The management of out of hospital cardiac arrest in Qatar

Case number ☐☐☐☐☐☐☐☐☐☐

Mode of Transportation

#1 Patient brought in by ☐1 EMS ☐2 Non-EMS

If 'Non-EMS', please specify ☐1 *Private ambulance* ☐2 *Own/Private transport* ☐3 *Public transport*

Incident Information

#2 Date of incident ☐☐☐☐☐☐☐☐ (dd/mm/yyyy)

#3 Location of incident **(Optional)**________________________________

(enter Zip/Postal code) ____________________ ☐☐☐☐☐☐ ☐ *Unknown*

#4 Location type
☐1 Home/residence ☐2 Healthcare facility ☐3 Public/Commercial building
☐4 Nursing home ☐5 Street/Highway ☐6 Industrial/work place
☐7 Transport center ☐8 Sports/recreation place ☐9 In EMS/Private ambulance
☐10 Other, specify ______________________________
☐11 Unknown ☐12 Not recorded

Patient Information

#5 Date of birth ☐☐☐☐☐☐☐☐ (dd/mm/yyyy) **Age** ☐☐☐ ☐ Days ☐ Months ☐ Years
☐1 Estimated ☐2 Unknown ☐3 Not recorded

#6 Gender ☐1 Male ☐2 Female ☐3 Unknown ☐4 Not recorded

#7 Race/nationality **(optional)**
☐1 Qatar ☐2 Gulf ☐3 other Arab ☐4 European ☐5 North American
☐6 Australian ☐7 India ☐8 Pakistan ☐9 Bangladesh ☐10 Philippine
☐11 Supra Saharan Africa ☐12 Sub-Saharan Africa ☐13 Nepal
☐14 Other,_________

#8 Medical history
O1 No O2 Unknown O3 Heart disease
O4 Diabetes O5 Cancer O6 Hypertension
O7 Renal disease O8 Respiratory disease O9 Hyperlipidemia
O10 Stroke O11 HIV O13 Not recorded
O12 Other, specify ____________________

Please tick the appropriate boxes and/or fill in the appropriate details. Version 25 July 2022

IRGC-07-SI-20-712 Validity: 12 03 2023 - 10 03 2024 E-stamped 12 Mar 2023

*Ability to perform daily activity independently: ☐1 Yes ☐2 No ☐3 Unknown ☐4 Not recorded

Patient supported by any ventricular assist device ☐1 Yes ☐2 No ☐3 Unknown ☐4 Not recorded

Patient has internal /external cardioverter-defibrillator ☐1 Yes ☐2 No ☐3 Unknown ☐4 Not recorded

Dispatch Information (Not Applicable for Non-EMS case)

***Dispatcher identified presence of arrest before EMS arrival?**

☐1 Yes ☐2 No ☐3 Unknown ☐4 Not recorded

***Dispatcher provided telephone CPR instructions to caller**

☐1 Yes ☐2 No ☐3 Unknown ☐4 Not recorded

#	Item	Time	Format
#9	**Time call received at dispatch center**	☐☐☐☐☐☐	(hh:mm:ss)
#10	**Time First responder dispatched**	☐☐☐☐☐☐	(hh:mm:ss)
#11	**Time Ambulance dispatched**	☐☐☐☐☐☐	(hh:mm:ss)
#12	**Time First responder arrived at scene**	☐☐☐☐☐☐	(hh:mm:ss)
#13	**Time Ambulance arrived at scene**	☐☐☐☐☐☐	(hh:mm:ss)
#14	**Time EMS arrived at patient side**	☐☐☐☐☐☐	(hh:mm:ss)
#15	**Time Ambulance left scene**	☐☐☐☐☐☐	(hh:mm:ss)
#16	**Time Ambulance arrived at ED**	☐☐☐☐☐☐	(hh:mm:ss)

☐ ***No* First Responder dispatched**

Prehospital Event and Resuscitation Information

#17 **Estimated time of arrest** ☐☐☐☐☐☐ (hh:mm:ss) ☐ *Unknown*

#18 **Arrest witnessed by**
- ☐1 Not witnessed
- ☐2 EMS/Private ambulance
- ☐3 Bystander – healthcare provider
- ☐4 Bystander – lay person
- ☐5 Bystander – family ☐6 Unknown ☐7 Not recorded

#19 **Bystander CPR** ☐1 Yes: ☐2 No ☐3 Unknown ☐4 Not recorded

If yes ☐1 Compression only ☐2 Ventilation only ☐3 Compression & ventilation

DA-CPR (Dispatcher-assisted CPR performed) ☐1 Yes ☐2 No ☐3 N/A ☐4 Unknown ☐5 Not recorded

IRGC-07-SI-20-712 Validity: 12 03 2023 - 10 03 2024 E-stamped 12 Mar 2023

#20 ***First*** **CPR initiated by**
☐1 No CPR initiated
☐2 First responder
☐3 Ambulance crew
☐4 Bystander – healthcare provider
☐5 Bystander – lay person
☐6 Bystander – family
☐7 Unknown ☐8 Not recorded

#21 **Bystander AED applied** ☐1 Yes ☐2 No ☐3 Unknown ☐4 Not recorded

#22 **Resuscitation attempted by EMS/Private ambulance** ☐1 Yes ☐2 No

#23 **First arrest rhythm** ☐1 VF ☐2 VT ☐3 PEA ☐4 Asystole
☐x Bradycardia ☐5 Unknown *shockable* rhythm
☐6 Unknown *unshockable* rhythm ☐7 Unknown ☐8 Not recorded

Changing Rhythm (Any two) ☐1 VF ☐2 PEA ☐3 Asystole ☐4 No ☐5 Unknown ☐6 Not recorded

#24 **Time CPR started by EMS/Private ambulance** ☐☐☐☐☐☐ (hh:mm:ss) ☐ *Unknown*

***Mechanism/Process in place to measure CPR quality** ☐ *Yes* ☐ *No* ☐ *Unknown* ☐ *Not recorded*

#25 **Time AED applied by EMS/Private ambulance** ☐☐☐☐☐☐ (hh:mm:ss) ☐ *Unknown*

#26 **Prehospital defibrillation** ☐1 Yes ☐2 No

If 'Yes', time of first shock given ☐☐☐☐☐☐ *(hh:mm:ss)* ☐ *Unknown* ☐ *Not recorded*

Number of shocks including shocks by public access defibrillators: N=________ ☐ *Unknown* ☐ *Not recorded*

#27 **Defibrillation performed by**
O1 First responder
O2 Ambulance crew
O3 Bystander – healthcare provider
O4 Bystander – lay person
O5 Bystander – family

***Vascular access type** ☐ *Central line* ☐ *Peripheral IV* ☐ *IO* ☐ *endotracheal* ☐ *Unknown* ☐ *Not recorded*

#28 **Mechanical CPR device used by EMS/Private ambulance** ☐1 Yes ☐2 No

(Or at any time during resuscitation) ***If 'Yes', please specify*** ☐1 *Load-Distributing Band*
☐2 *Active Compression Decompression* ☐3 *Mechanical Piston*
☐4 *Other* ☐5 *Unknown* ☐6 *Not recorded*

Institutional Review Board Hamad Medical Corporation · Doha · Qatar Hamad

IRGC-07-SI-20-712 Validity: 12 03 2023 - 10 03 2024 E-stamped 12 Mar 2023

#29 Prehospital advanced airway ☐1 Yes ☐2 No

If 'Yes', please specify ☐1 *Oral/Nasal ET* ☐4 *King airway*

☐2 *Combitube* ☐3 *LMA* ☐4 *Surgical airway* ☐5 *Multiple*

☐6 *Other* ☐7 *Unknown* ☐8 *Not recorded*

#30 Prehospital drug administration ☐1 Yes ☐2 No

If 'Yes', select drugs given O1 *Epinephrine (dose:)* O2 *Atropine*

O3 *Amiodarone* O4 *Bicarbonate* O5 *Lidocaine* O6 *Dextrose*

O7 *Other* O8 *Unknown* O9 *Not recorded*

\# **Drug Timings: time 1st drug given** ☐☐☐☐☐☐ *(hh:mm:ss)* ☐ *Unknown* ☐ *Not recorded*

#31 Return of spontaneous circulation at scene/en-route ☐1 Yes ☐2 No

If 'Yes', specify time ☐☐☐☐☐☐ *(hh:mm:ss)* ☐ *Unknown* ☐ *Not recorded*

\# Presence of STEMI at time of first ECG after ROSC ☐ Yes ☐ No ☐NA ☐ Unknown ☐ Not recorded

\# ***Pupil Reactivity on ROSC*** ☐ Yes ☐ No ☐ Unknown ☐ Not recorded

#32 CPR discontinued at scene/en-route ☐1 Yes ☐2 No

If 'Yes', please specify ☐1 *DNAR*

☐2 *ROSC*

☐3 *Medical control order*

☐4 *Obvious signs of death*

☐5 *Protocol/policy requirements completed*

Disposition

#33 Final status at scene ☐1 Conveyed to ED ☐2 Pronounced dead at scene

#34 Cause of arrest ☐1Trauma ☐2 Non-trauma

If 'Non-trauma', please specify ☐1 *Presumed cardiac etiology* ☐2 *Respiratory/ Asphyxia*

☐3 *Electrocution* ☐4 *Drowning* ☐6 *Drug overdose* ☐5 *Other*__________________

#35 Level of destination hospital ☐1 Tertiary ☐2 Community

#36 Destination hospital ☐1 HGH ☐2 HMGH ☐3 AWH ☐4 AKH ☐5 HH ☐6 Cuban H

☐7 Other__________________

Institutional Review Board Hamad Medical Corporation · Doha · Qatar Hamad

IRGC-07-SI-20-712 Validity: 12 03 2023 - 10 03 2024 E-stamped 12 Mar 2023

#37 Patient's status at ED arrival
☐1 ROSC
☐2 Ongoing resuscitation
☐3 Transported without resuscitation

ED Resuscitation Information (Not Applicable for cases that were pronounced dead at scene)

#38 Date of arrival at ED ☐☐☐☐☐☐☐☐ (dd/mm/yyyy)

#39 Time of arrival at ED ☐☐☐☐☐☐ (hh:mm:ss)

#40 Patient status on arrival at ED
Pulse ☐1 Yes ☐2 No ☐ *Unknown* ☐ *Not recorded*
Breathing ☐1 Yes ☐2 No ☐ *Unknown* ☐ *Not recorded*

#41 Cardiac rhythm on arrival at ED ☐1 VF ☐2 VT ☐3 PEA
☐ *Unknown* ☐ *Not recorded* ☐4 Asystole ☐5 Sinus or other perfusing rhythm

\# **Changing Rhythm (Any two)** ☐1VF ☐2 PEA ☐3 Asystole ☐4 No ☐5 *Unknown* ☐6 *Not recorded*

#42 ED defibrillation performed ☐1 Yes ☐2 No

\# ***Number of shocks delivered: N=___________*** ☐ *Unknown* ☐ *Not recorded*

\# ***Vascular access type** ☐ *Central line* ☐ *Peripheral IV* ☐ *IO* ☐ *endotracheal* ☐ *Unknown* ☐ *Not recorded*

#43 Mechanical CPR device used at ED ☐1 Yes ☐2 No ☐ *Unknown* ☐ *Not recorded*
If 'Yes', please specify ☐1 *Load-Distributing Band* ☐3 *Mechanical Piston*
☐2 *Active Compression Decompression* ☐4 *Other*

#44 Advanced airway used at ED ☐1 Yes ☐2 No
If 'Yes', please specify ☐1 *Oral/Nasal ET* ☐2 *Combitube* ☐3 *LMA*
☐4 *King airway* ☐5 *Surgical airway* ☐6 *Multiple* ☐7 *Other_____________*
☐8 *Unknown* ☐9 *Not recorded*

#45 Drug administered at ED ☐1Yes ☐2 No
If 'Yes', select drugs given O1 *Epinephrine* O4 *Bicarbonate* O7 *Other*
Total Epinephrin given mg (AMS&ED): O2 *Atropine* O5 *Lidocaine* O8 *Unknown*
O3 *Amiodarone* O6 *Dextrose* O9 *Not recorded*

IRGC-07-SI-20-712 Validity: 12 03 2023 - 10 03 2024 E-stamped 12 Mar 2023

#46 **Return of spontaneous circulation at ED** ☐1 Yes ☐2 No ☐3 NA

If 'Yes', specify time ☐☐☐☐☐☐ *(hh:mm:ss)* ☐ *Unknown*

Pupil Reactivity <u>on ROSC</u> ☐ Yes ☐ No ☐ Unknown ☐ Not recorded ☐*NA*

<u>12 lead ECG after ROSC</u> ☐ Yes ☐ No ☐ Unknown ☐ Not recorded ☐ NA

Presence of STEMI at time of first ECG after ROSC ☐ Yes ☐ No ☐ Unknown ☐ Not recorded ☐ NA

*Coronary reperfusion attempted ☐ Yes ☐ No ☐ Unknown ☐ Not recorded

Type of reperfusion: ☐ Thrombolysis ☐ Angiography only ☐ PCI ☐ CABG ☐ NA

Timing of reperfusion: ☐ intra-arrest ☐ within 24h of ROSC ☐>24h but before discharge
☐ Unknowing ☐ Not recorded ☐ NA

#47 **Emergency PCI performed** ☐1 Yes ☐2 No

#48 **Emergency CABG performed** ☐1 Yes ☐2 No

#49 **TTM Hypothermia therapy initiated** ☐1 Yes ☐2 No

If 'Yes', specify time ☐☐☐☐☐☐ *(hh:mm:ss)* ☐ *Unknown* ☐ *Not recorded*

Target teperature:____________C ☐ *Unknown* ☐ *Not recorded*

Setting where TTM initiated: ☐ *Intra-arrest* ☐ *post-ROSC prehospital* ☐ *post-ROSC in-hospital*
☐ *TTM indicated but not performed* ☐ *TTM not indicated* ☐ *Unknown* ☐ *Not recorded*

*Targetted oxygenation/ventillation <u>after ROSC</u>: ☐ O2 & CO2 ☐ O2 only ☐ CO2 only ☐ not used
☐ Unknown ☐ Not recorded ☐ NA

Targeted BP management ☐ mmHg__________ ☐ No target set ☐ Unknown ☐ Not recorded ☐ NA

#50 **ECMO therapy initiated** ☐1 Yes ☐2 No ☐3 Unknown ☐4 Not recorded

If yes: ☐ before ROSC ☐ after ROSC

***Supplemental Post resuscitation process**:

1- IABP: ☐Yes ☐ No ☐ Unknown ☐ Not recorded

2-PH: ☐ venous Value______ ☐ art. Value______ ☐ Unknown ☐ Not recorded

3-Lactate: ☐ venous Value______ ☐ art. Value______ ☐ Unknown ☐ Not recorded

4-Glucose titrated to specific target <u>after ROSC</u>: ☐ Yes ☐ No ☐ NA ☐ Unknown ☐ Not recorded

5-Neurological tests: ☐ Clinical examination ☐ SSEP ☐ NSE ☐ CT brain ☐ MRI brain
☐ Other, specify ____

Did the test lead to discontinuation of treatment? ☐ Yes ☐ No ☐ Unknown ☐ Not recorded

Institutional Review Board Hamad Medical Corporation · Doha · Qatar
IRGC-07-SI-20-712 Validity: 12 03 2023 - 10 03 2024 E-stamped 12 Mar 2023

#51 **Cause of arrest**		☐1 Trauma	☐2 Non-trauma
	If 'Non-trauma', please specify	☐1 *Presumed cardiac etiology*	☐2 *Respiratory/ Asphyxia*
☐3 *Electrocution* ☐4 *Drowning*	☐6 *Drug overdose*	☐5 *Other*__________________	☐7 *Not recorded*
#52 **Reason for discontinuing CPR at ED**		☐1 Death	☐3 ROSC
		☐2 DNAR	☐4 ECMO therapy
#53 **Outcome of patient**	☐1 Admitted		☐3 Died in ED
	☐2 Transferred to another hospital		☐4 Unknown ☐5 *Not recorded*

Hospital Outcome ***(FOR PATIENT WHO SURVIVED TO ADMISSION)***

#54 **Patient status**
- ☐1 Discharged alive
- ☐2 Remains in hospital at 30th day post arrest
- ☐3 Died in hospital

#55 **Date of Discharge or Death** ☐☐☐☐☐☐☐☐ (dd/mm/yyyy)

Time of Discharge or Death ☐☐☐☐☐☐☐☐ (hh/mm/ss)

***Cause of death** (in medical record/death certificate): ______________________________

#56 **Patient neurological status on discharge or at 30th day post arrest**
- Cerebral Performance Category ☐
- Overall Performance Category ☐
- ☐ Unknown

Neurological outcome measured ☐ face to face ☐ Phone FU ☐ extracted from notes ☐ combination

Patient Health and Quality of Life ***(FOR PATIENT WHO IS DISCHARGED ALIVE or ALIVE ON 30th DAY POST ARREST)***

EQ-5D Health Dimensions

#57	Mobility	☐1 No problem	☐2 Some problems	☐3 Confined to bed
#58	Self-care	☐1 No problem	☐2 Some problems	☐3 Unable to wash or dress
#59	Usual activities	☐1 No problem	☐2 Some problems	☐3 Unable to perform
#60	Pain/Discomfort	☐1 None	☐2 Moderate	☐3 Extreme
#61	Anxiety/Depression	☐1 None	☐2 Moderate	☐3 Extreme

Institutional Review Board Hamad Medical Corporation · Doha Qatar

IRGC-07-SI-20-712 Validity: 12 03 2023 - 10 03 2024 E-stamped 12 Mar 2023

#62 **EQ-5D Visual Analog Scale (VAS)**

100 — 90 — 80 — 70 — 60 — 50 — 40 — 30 — 20 — 10 — 0

**100 (best imaginable health state) and 0 (worst imaginable health state)*

Supplemental Outcomes:

Supplemental Outcomes

Survival at 12 months after cardiac arrest ☐ Yes ☐ No ☐ Unknown ☐ Not recorded

**Transported to hospital* ☐ Yes_______________________ ☐ No ☐ Unknown ☐ Not recorded

**Patient-reported outcomes measures (free text):*

**Organ Donation (non-survivors):* ☐*Yes* _______________ ☐ *No* ☐ Unknown ☐ Not recorded

Patient Health and Quality of Life *(FOR PATIENT WHO IS ALIVE ON 12 months POST ARREST)*

EQ-5D Health Dimensions

#	Mobility	☐1 No problem	☐2 Some problems	☐3 Confined to bed
#	Self-care	☐1 No problem	☐2 Some problems	☐3 Unable to wash or dress
#	Usual activities	☐1 No problem	☐2 Some problems	☐3 Unable to perform
#	Pain/Discomfort	☐1 None	☐2 Moderate	☐3 Extreme
#	Anxiety/Depression	☐1 None	☐2 Moderate	☐3 Extreme

EQ-5D Visual Analog Scale (VAS)

100 — 90 — 80 — 70 — 60 — 50 — 40 — 30 — 20 — 10 — 0

**100 (best imaginable health state) and 0 (worst imaginable health state)*

# Patient neurological status at 12 months post arrest:

Cerebral Performance Category ☐ Overall Performance Category ☐ Unknown ☐

# Neurological outcome measured ☐ face to face ☐ Phone FU ☐ extracted from notes ☐ combination

Notes:

Institutional Review Board · Hamad Medical Corporation · Doha · Qatar · Hamad

IRGC-07-SI-20-712 Validity: 12 03 2023 - 10 03 2024 E-stamped 12 Mar 2023
